# Photoreduction of CO_2_ on TiO_2_/SrTiO_3_ Heterojunction Network Film

**DOI:** 10.1186/s11671-015-1054-5

**Published:** 2015-08-28

**Authors:** Yongsheng Bi, Lanlan Zong, Chen Li, Qiuye Li, Jianjun Yang

**Affiliations:** Key Laboratory for Special Functional Materials, Henan University, Kaifeng, 475004 China

**Keywords:** Nanotube titanic acid, Porous network film, TiO_2_/SrTiO_3_ heterojunction, CO_2_ photoreduction, Product selectivity

## Abstract

Nanotube titanic acid (NTA) network film has a porous structure and large BET surface area, which lead them to possessing high utilization of the incident light and strong adsorption ability. We used NTA as the precursor to fabricate a TiO_2_/ SrTiO_3_ heterojunction film by the hydrothermal method. In the process of the reaction, part of NTA reacted with SrCl_2_ to form SrTiO_3_ nanocubes, and the remainder dehydrated to transform to the rutile TiO_2_. The ratio of TiO_2_ and SrTiO_3_ varied with the hydrothermal reaction time. SEM and TEM images indicated that SrTiO_3_ nanocubes dispersed uniformly on TiO_2_ film, and the particle size and crystallinity of SrTiO_3_ nanocubes increased with the reaction time prolonging. The TiO_2_/SrTiO_3_ heterojunction obtained by 1 h showed the best activity for CO_2_ photoreduction, where the mole ratio of TiO_2_ and SrTiO_3_ was 4:1. And the photo-conversion efficiency of CO_2_ to CH_4_ improved remarkably after the foreign electron traps of Pt and Pd nanoparticles were loaded. The highest photocatalytic production rate of CH_4_ reached 20.83 ppm/h cm^2^. In addition, the selectivity of photoreduction product of CO_2_ was also increased apparently when Pd acted as the cocatalyst on TiO_2_/SrTiO_3_ heterojunction film.

## Background

Nowadays, the fossil fuels are still the main energy resource for our society. However, the shortage of fossil fuels and the growing environmental concerns due to the emission of large amounts of CO_2_ during the combustion of fossil fuels have become the global problems. Conversion of CO_2_ into useful hydrocarbon fuels is a possible avenue to develop alternative fuels, and prevent the green house effect on the global temperature. For example, the chemical conversion of CO_2_ into industrially beneficial compounds is advantageous in terms of green and sustainable chemistry because CO_2_ is an inexpensive, nontoxic and abundant C1 feedstock [1]. Particularly, catalytic conversion of CO_2_ to hydrocarbon fuels and chemicals have attracted much attention in recent years [2–6].

At the same time, TiO_2_-based materials are the most common photocatalysts because of their many advantages. Especially, one-dimensional TiO_2_ nanostructures have become of increasing importance in applications of photocatalysis, photoelectron-chemical process, and dye-sensitized solar cells due to their superior properties in comparison with other TiO_2_ nanostructured counterparts [7–12]. Besides, TiO_2_-based nanomaterials, especially the layered titanate nanotubes, obtained by the hydrothermal method possess the large BET surface area, strong ion-exchange capacity, and strong adsorption ability [13]. The high recombination of the photo-generated charge carriers leads to the low photocatalytic activity of TiO_2_-based nanomaterials. In order to overcome this drawback, forming a heterojunction structure by combing TiO_2_ with another semiconductor is considered to be one of the efficient ways to suppress the recombination of the photo-excited electron-hole pairs and to enhance the photocatalytic efficiency [14, 15]. SrTiO_3_ with the perovskite structure is one of semiconductors with a flat band potential lower than that of TiO_2_, and it is easily to be formed a heterojunction structure with TiO_2_ in the preparation process [16–19]. In this regard, the photo-generated electrons would centralize on the conduction band of TiO_2_, and the holes would concentrate on the valence band of SrTiO_3_ under UV light irradiation, and as a result, the recombination efficiency of the photo-generated charge carriers is inhibited, and thereby the photocatalytic activity would be improved [20].

On the basis of above consideration, we intent to fabricate the TiO_2_/SrTiO_3_ heterojunction structure film by the hydrothermal method. Herein, the cubic SrTiO_3_ was achieved by hydrothermal treatment of the orthorhombic titanic acid in SrCl_2_ aqueous solution by adjusting pH = 13. Notably, by simply tuning reaction time, the crystallinity, morphology and the amount of SrTiO_3_ nanostructures can be controlled easily. The TiO_2_/SrTiO_3_ heterostructure film exhibited the good photocatalytic performance for CO_2_ photoreduction. In order to further improve the transformation yield, the foreign electron traps of Pt and Pd nanoparticles were loaded on the film by the photoreduction approach. The relationship between the photocatalytic properties of TiO_2_/SrTiO_3_ heterostructure film with their morphology and structure was investigated systematically.

## Methods

### Preparation of the Film Photocatalysts

Ti foil with a size of 2 cm × 4 cm was put into an autoclave containing a concentrated 10 M NaOH aqueous solution, and then reacted at 120 °C for 24 h. After cooling down, the obtained film was washed with distilled water several times, and then immersed in 0.1 M HCl aqueous solution for 12 h to obtain the titanic acid nanotubes film (TAN). After that, TAN was put into an autoclave containing 80 mL 0.05 M SrCl_2_ aqueous solution, and the pH value of the solution was adjusted to 13 by NaOH solution. The autoclave was kept at 120 °C for 1 h, 2 h, and 3 h respectively. The as-fabricated films were washed with deionized water several times, and then dried with the stream of N_2_. The samples obtained in the different reaction time were denoted as TS1, TS2, and TS3, respectively. In order to further increase the photocatalytic performance, the foreign electron traps of Pt and Pd nanoparticles were deposited on the TS1 surface by photoreduction of H_2_PtCl_6_ and PdCl_2_ solution under the irradiation of the high-pressure mercury lamp for 1 h. The obtained products were denoted as TS1-Pt and TS1-Pd.

### Characterization

X-Ray powder diffraction (XRD) patterns of the films were measured on a Philips X’Pert Pro X-ray diffractometer (Holland) (Cu *K*α radiation; 2*θ* range 5 ~ 70°, step size 0.08°, time per step 1.0 s, accelerating voltage 40 kV, and applied current 40 mA). The morphologies of the samples were taken on SEM (JSM-7100 F, JEOL Co., Japan) and TEM (JEM-2010, JEOL Co., Japan). X-ray photoelectron spectra (XPS) were recorded with a Kratos AXIS Ultra spectrometer (excitation source: monochromatized Al *K*α (*hν* = 1486.6 eV); voltage 15 kV, current 10 mA). And the C 1 s binding energy of hydrocarbon (284.8 eV) was used as the standard for the correction of charging shift.

### Evaluation of Photocatalytic Activity

The photocatalytic reduction of CO_2_ was conducted in a flat closed reactor with the inner capacity of 358 mL containing 20 mL 0.1 mol/L KHCO_3_ solution. The prepared samples were located in the center of the reactor and then the ultra-pure gaseous CO_2_ and water vapor was flowed through the reactor for 2 h to achieve the adsorption-desorption equilibrium. Before illumination, the reactor was sealed. The light source was the high pressure Hg lamp with 300 W, and the intensity of the incident light was measured to be 10.4 mW/cm^2^. Both sides of the Ti foil have transformed to TiO_2_/SrTiO_3_ heterojunction film, but only one side under the light irradiation took part in the CO_2_ photo-reduction reaction. The photocatalytic reaction was typically performed at room temperature for 6 h. The concentration of CO, CO_2_, and CH_4_ were measured by a gas-chromatography (GC). Moreover, the electrochemical impedance spectroscopy (EIS) properties were measured in 0.05 M Na_2_SO_3_ aqueous solution using a three-electrode photoelectrochemical cell with TS film as the working electrode, an Ag/AgCl electrode as the reference, and a platinum meshwork as the counter electrode.

## Results and Discussion

### Phase Structure of the Porous Film

**Fig. 1:**
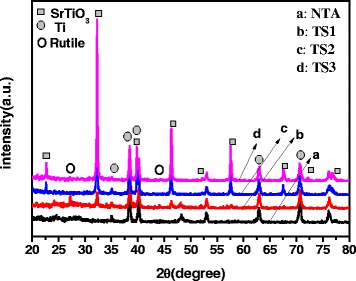
XRD patterns of film photocatalysts

The phase structure of the films was measured by the XRD technique. As illustrated in Fig. 1, curve *a* showed that the titanic acid nanotube (TAN) film belongs to the orthorhombic structure, which is consistent with our previous work [21]. There are some characteristic peaks at 38.5°, 40.2°, 63.1°, and 70.7°, which were indexed to the metallic Ti. That indicated only the surface of the Ti foil transformed to TAN after reacting with NaOH, and the interior part still remained as Ti metal. After hydrothermal treatment of TAN film in SrCl_2_ solution at 120 °C for 1 h, additional diffraction peaks at 22.7°, 32.2°, 46.3°, 57.6°, and 67.8° appeared, which were corresponded to the (100), (110), (200), (211), and (220) crystal planes of cubic SrTiO_3_, respectively (as shown in curve *b*). This result indicated that part of TAN successfully converted into the cubic SrTiO_3_. At the same time, two peaks at about 27.46° and 44.08° of rutile TiO_2_ appeared, indicating that the residue TAN converted to rutile in the base solution. When the reaction time prolonged to 2 h, the peaks of TiO_2_ disappeared, that illustrated TAN transformed into SrTiO_3_ completely (as shown in curve *c*). As the increase of the reaction time, the peak intensity of SrTiO_3_ increased by comparing curve b and c, which indicated that the crystallinity of the cubic SrTiO_3_ improved.

### Morphology and Composition Analysis of TiO_2_/SrTiO_3_ Heterostructure

**Fig. 2:**
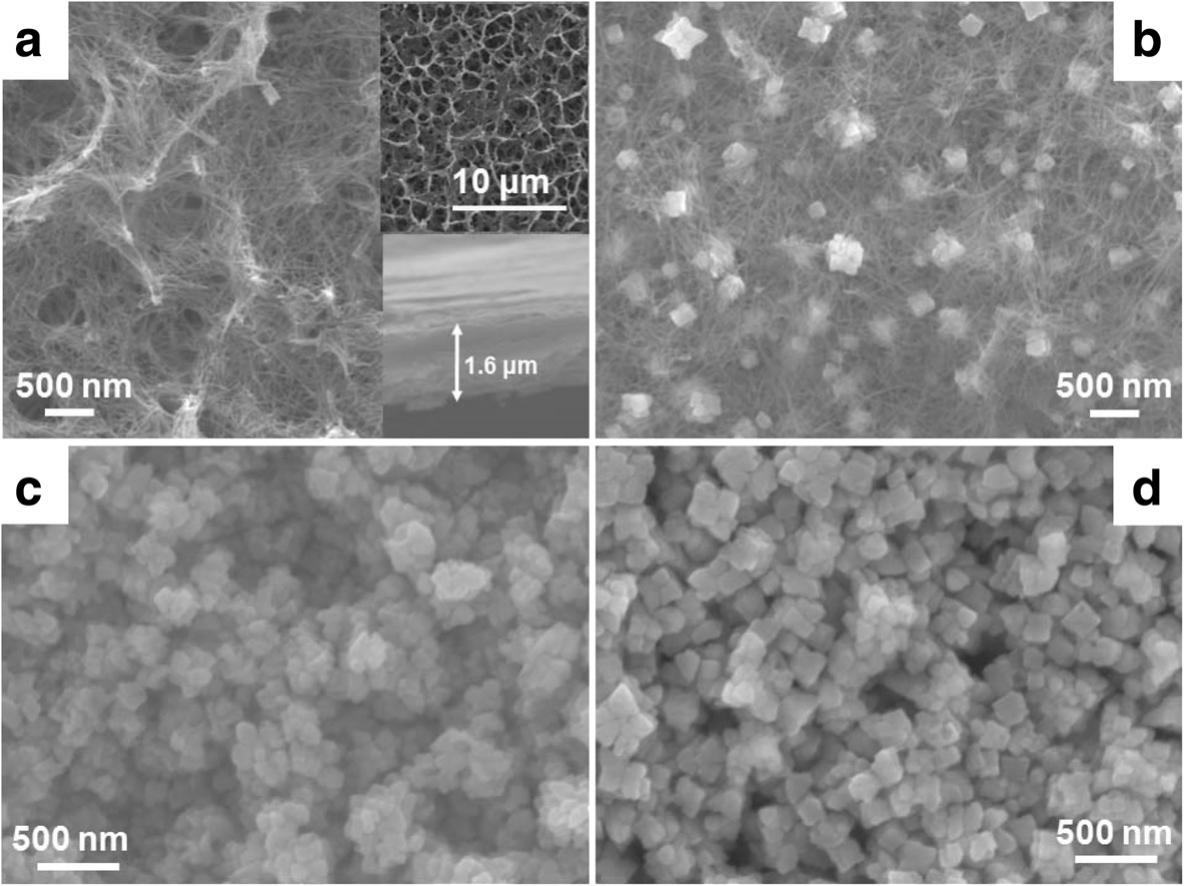
The top SEM images of the film photocatalysts. **a** NTA. **b** TS1. **c** TS2. **d** TS3. The inset of Fig. 2a shows the large-scale image and the thickness of the film

**Fig. 3:**
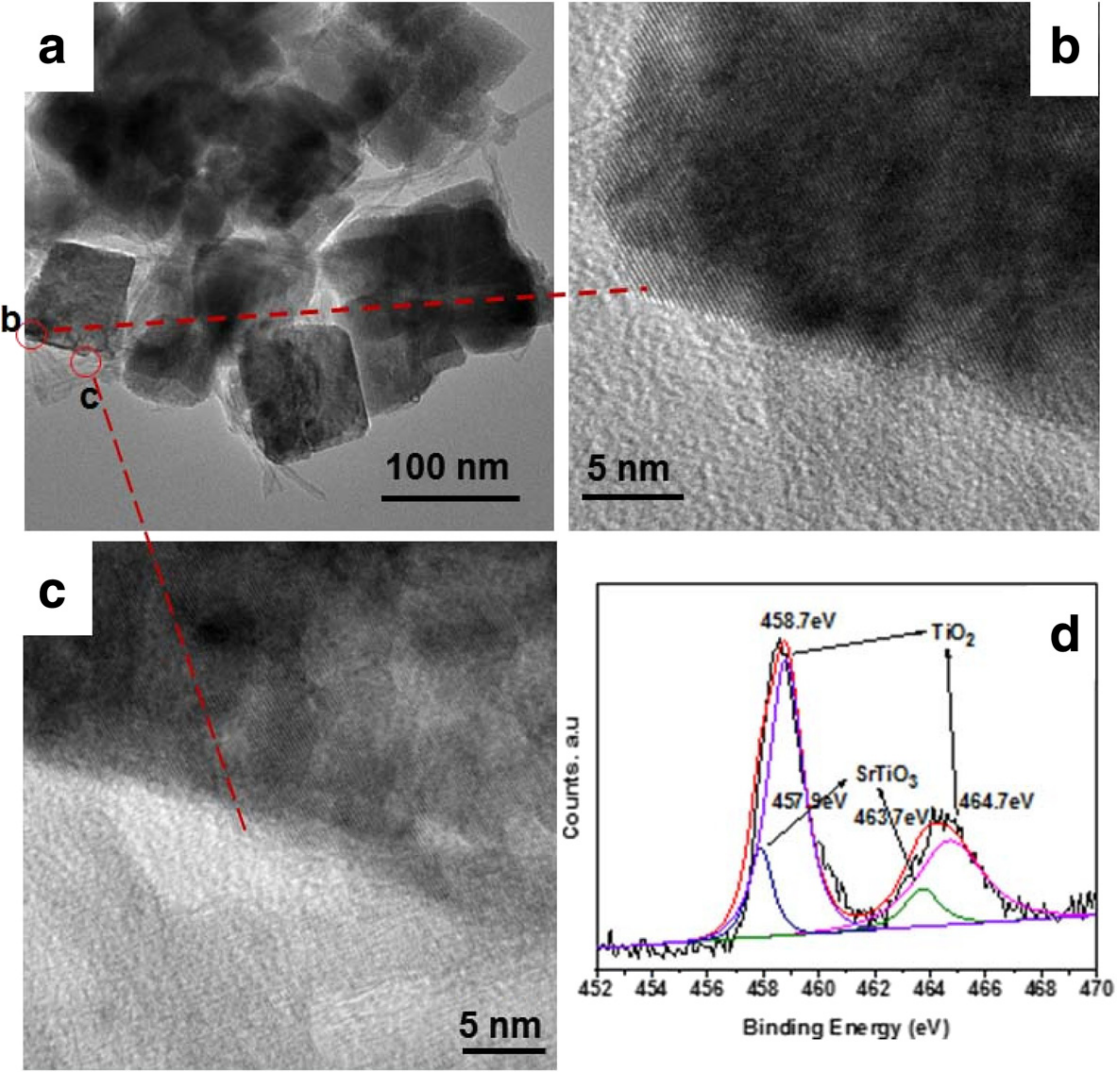
TEM and HRTEM images TiO_2_/SrTiO_3_ heterojunction (**a**-**c**), XPS spectrum of Ti 2p in TS1 film (**d**)

Figure 2 shows the FE-SEM images of TAN and TiO_2_/SrTiO_3_ heterostructure films. From Fig. 2a, we can see that TAN film consisted of a large amount of nanotubes, and many nanotubes intertwined together to form a porous an incompact structure. The diameters of TAN nanotubes were uniform, and their lengths expanded to several micrometers. The inset figure showed that the thickness of TAN film was about 1.6 μm. Figure 2b illustrated that some cubic nanoparticles emerged in TAN films, indicated that part of TAN has transformed to SrTiO_3_. As the increase of the reaction time, TAN disappeared and transformed to SrTiO_3_ nanoparticles completely (shown in Fig. 2c). When the reaction time increased to 3 h, the irregular SrTiO_3_ nanoparticles grew to the regular SrTiO_3_ nanocubes, indicating that the crystallinity becomes better. The above results were in accordance very well with the XRD results. And the average particle size of SrTiO_3_ nanocubes in Fig. 2d was about 70–80 nm. To further observe the morphology of TiO_2_/SrTiO_3_ heterojunction strucuture, some powders were peeled off from the film. The TEM images in Fig. 3 showed that, TiO_2_ nanotubes and SrTiO_3_ nanocubes co-existed in the TiO_2_/SrTiO_3_ heterojunction strucuture. The diameter of TiO_2_ nanotubes was about 8–10 nm, and SrTiO_3_ nanocubes were ca. 80 nm, which consisted with the SEM results. From Fig. 3b, we can obviously found that the figure lattice spacing of SrTiO_3_ nanocubes was regular and clear, indicating that the crystallinity of SrTiO_3_ was very good. And Fig. 3c showed that TiO2 nanotubes and SrTiO_3_ nanocubes formed a very closely heterjunction structure, which should be favorable for the separation of the charge carriers. The phase composition of TiO_2_/SrTiO_3_ heterojunction was also measured by XPS techniques (shown in Fig. 3d). The XPS spectrum of Ti 2p was wide and asymmetric, which indicated that there could be more than one chemical state according to the binding energy [20, 22]. Using the XPS Peak fitting program, the Ti 2p XPS spectrum could be fitted to two kinds of chemical states, that was ascribed to Ti^4+^/TiO_2_ and Ti^4+^/SrTiO_3_, respectively [23]. The mole ratio of TiO_2_ and SrTiO_3_ was tested to be 4:1.

### Photoreduction of CO_2_ on TiO_2_/SrTiO_3_ Heterojunction Films

**Fig. 4:**
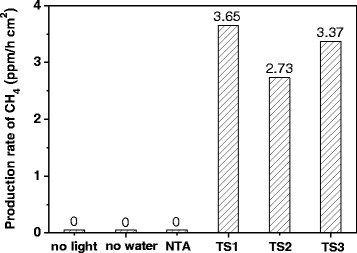
Photocatalytic production rate of CH_4_ on different photocatalyts

The photo-reduction reaction of CO_2_ was selected to evaluate the photocatalytic activity of TiO_2_/SrTiO_3_ heterojunction films. As shown in Fig. 4, the production rate of CH_4_ on TS1, TS2, and TS3 films was 3.67, 2.73, and 3.37 ppm/h · cm^2^, respectively. From the above XRD and SEM result, we knew that TS1 was TiO_2_/SrTiO_3_ heterojunction films, and the main composition of TS2 and TS3 was SrTiO_3_. These results indicated that the TiO_2_/SrTiO_3_ heterojunction exhibited the best photocatalytic activity of CO_2_ photoreduction to CH_4_. The reasons of the high photo-activity of TiO_2_/SrTiO_3_ film can be attributed to three aspects. Firstly, the efficient heterojunction by direct coupling of TiO_2_ and SrTiO_3_ nanostructures during short duration hydrothermal treatment caused the Fermi level to equilibrate and reduced the recombination of charge carriers at the surface of the heterostructure. And thus favored the separation of photogenerated electrons-holes pairs and improved the photo-conversion efficiency [24]. Secondly, the large BET surface areas and strong adsorption capability of the TiO_2_/SrTiO_3_ network structure would facilitate more CO_2_ molecules to adsorb, and thus the localized concentration of CO_2_ molecules would be higher, which would enhance the photoreduction reaction rate of CO_2_ to methane. Thirdly, more light can be scattered or reflected in the porous and incompact structure of the TiO_2_/SrTiO_3_ network film, so the utilization yield of the irradiated light would be improved [25]. Comparison the photoactivity of TS2 and TS3, we found that the photoreduction rate of CO_2_ on TS3 was higher, which should be due to the high crystallinity of TS3 nanocubes.

**Fig. 5:**
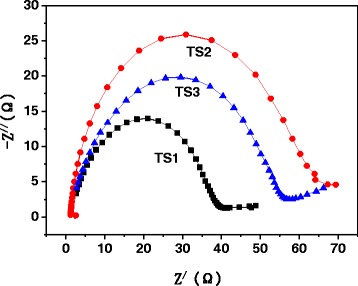
EIS Nyquist plots of TS1, TS2, and TS3 electrodes under light irradiation

As an effective tool for probing the features of surface-modified electrodes, electrochemical impedance spectroscopy (EIS) was employed to analyze the electron transport properties of TiO_2_/SrTiO_3_ (TS) electrodes. As shown in Fig. 5, the impedance arc radius of TS1 was much smaller than TS2 and TS3, and that of TS2 was the largest. EIS spectrum often displays the conductivity of an electrode, and a larger arc radius usually illustrates a higher charge transfer resistance [26–28]. So, The EIS results indicated that the separation and transfer efficiency of the photo-generated charge carriers of TS1 was much higher than that of TS2 and TS3, which is consistent very well with their photocatalytic activity.

To confirm the real photocatalytic reduction process of CO_2_ to CH_4_ on TiO_2_/SrTiO_3_ heterojunction films, some related reference experiments were conducted. When the reaction was preceded in dark, there was no CH_4_ detectable, indicating that the photo-excited process of TS film was essential in the photo-reduction of CO_2_. When the experiment was conducted in the absence of H_2_O, almost no CH_4_ was produced. That should be due to no reduce species (H^+^) took part in the photo-reduction of CO_2_. If we use the NTA nanotube film to replace TS film, there was also no photoactivity. The above comparison experiments illustrated that the conversion of CO_2_ to CH_4_ on TS films was indeed the photo-reduction process.

### Photocatalytic Activity on Pt (or Pd) Loaded TiO_2_/SrTiO_3_ Heterojunction Films

**Fig. 6:**
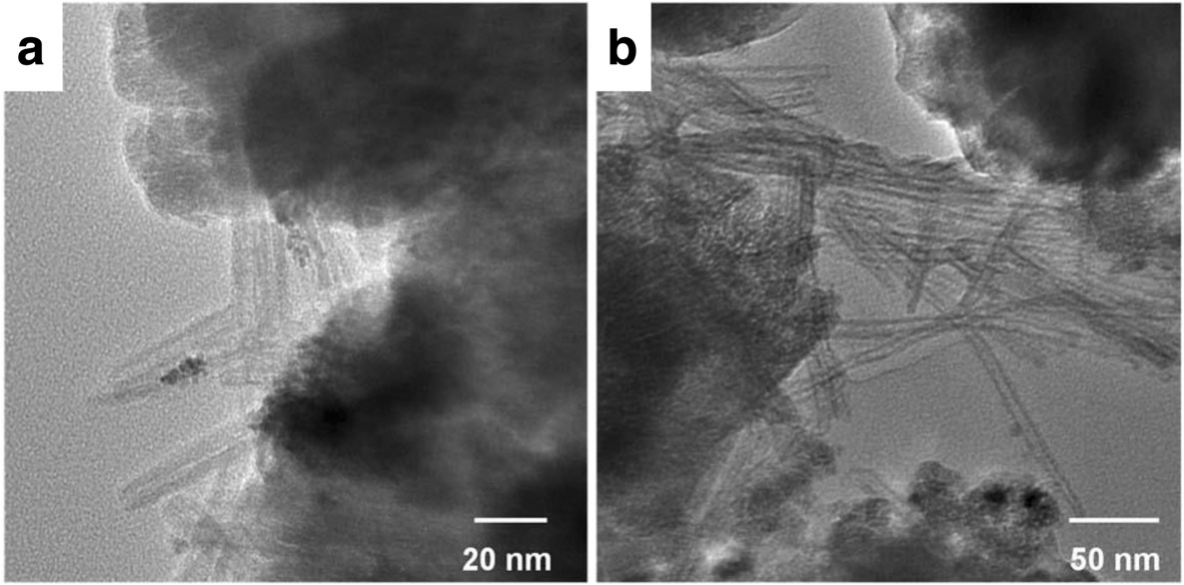
TEM images of Pt loaded TS1 (**a**) and Pd loaded TS1 (**b**)

**Fig. 7:**
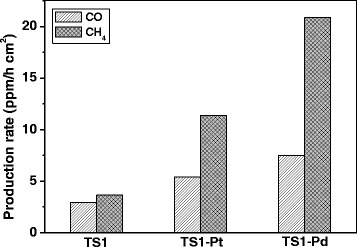
Photocatalytic production rate of CH_4_ and CO on TS1, TS1-Pt, and TS1-Pd

In order to further raise the photocatalytic activity of TS film, Pt and Pd nanoparticles were loaded on TS1 film by the photoreduction method. As shown in Fig. 6, Pt and Pd nanoparticles with the average size of 4–6 nm dispersed uniformly on TiO_2_ nanotubes. Due to the similar size of Pt (or Pd) nanoparticles and the TiO_2_ nanotube diameters, so it is easily to observe and distinguish the loaded noble metals. However, the particle size of SrTiO_3_ nanocubes is about 70–80 nm, and the crystallinity of SrTiO_3_ in TS1 is not very good, and thus the borderline of SrTiO_3_ nanocubes is not clear, so it is difficult to differentiate the loaded small Pt and Pd nanoparticles. Because Pt and Pd nanoparticles were loaded on TS1 by the photoreduction, they should be dispersed on both TiO_2_ nanotubes and SrTiO_3_ nanocubes. The activity of CO_2_ photo-reduction on Pt or Pd loaded TiO_2_/SrTiO_3_ heterojunction films were illustrated in Fig. 7. Compared with the bare TS1 film, the photocatalytic activity for CH_4_ and CO production after loading Pt or Pd nanoparticles increased remarkably. The production rate of CH_4_ increased from 3.67 to 11.37 and 20.83 ppm/h cm^2^ when Pt and Pd loaded on TS1. In the meantime, the production rate of CO increased from 2.93 to 5.38 and 7.49, respectively. Pt or Pd nanoparticles are often used as the co-catalysts to increase the separation efficiency of the photo-generated electron-hole pairs [29, 30]. In this work, they indeed played the important role to enhance the photocatalytic activity for CO_2_ reduction. In addition, comparison TS1-Pt and TS1-Pd film, we found that the loaded noble metals played different effect on the enhancement of the photoactivity. When Pt loaded TS1, the production rate of CH_4_ increased 2.1 times. While for Pd loaded TS1, it increased 4.7 times, indicating that Pd is more efficient to improve the photoactivity of CH_4_ production. Moreover, the production rate of CH_4_ and CO increased 4.7 times and 1.6 times on TS1-Pd than TS1, implying that loading Pd nanoparticles on TS1 film is favorable to improve the selectivity of CO_2_ phtoreduction to CH_4_.

## Conclusions

In summary, TiO_2_/SrTiO_3_ heterojunction network films were prepared successfully by hydrothermal method using nanotube titanic acid film (NTA) as the precursor. In the basic reaction process, part of NTA reacted with SrCl_2_ to form SrTiO_3_ naocubes, and the residues transformed to rutile TiO_2_. As prolonging the reaction time to 2 h, NTA transformed to SrTiO_3_ naocubes completely. The TiO_2_/SrTiO_3_ heterojunctions obtained at 1 h exhibit the best photocatalytic performance for the photoreduction of CO_2_. The increased photocatalytic activity can be responded by the enhanced charge separation derived from the coupling effect the TiO_2_ and SrTiO_3_ components, large surface area (BET) and strong adsorption ability of TS1 network porous film. In addition, the photoreduction activity of CO_2_ to CH_4_ increased from 3.67 to 11.37 and 20.83 ppm/h cm^2^ when Pt and Pd loaded on TS1 film. Especially, Pd also played the important role to increase the selectivity of photoreduction CO_2_ to CH_4_.
